# Supplementary material for: Revised oceanic molybdenum isotope budget from deep-sea pelagic sediments
Source: Nat Commun. 2025 Nov 18;16:10086. doi: 10.1038/s41467-025-65006-5 (PMC12627635; doi:10.1038/s41467-025-65006-5)
Supplement: Supplementary file 1 — Supplementary Information [file 41467_2025_65006_MOESM1_ESM.pdf]

Supplementary Information for

Revised Oceanic Molybdenum Isotope Budget from Deep-Sea Pelagic Sediments

Zhibing Wang[1,2*], Jie Li[1], Bangqi Hu[3], Liang Zou[3*], Xue Ding[3], Le Zhang[1], Jinlong Ma[1], Gangjian Wei[1,2]

1. State Key Laboratory of Isotope Geochemistry, Guangzhou Institute of Geochemistry, Chinese Academy of Sciences, Guangzhou 510640, China
2. College of Earth and Planetary Sciences, University of Chinese Academy of Sciences, Beijing 100049, China
3. Qingdao Institute of Marine Geology, China Geological Survey, Qingdao, China

*Corresponding author: Zhibing Wang
E-mail address: wangzhibing@gig.ac.cn

*Corresponding author: Liang Zou
E-mail address: zouliang04@163.com

S1. Calculating Authigenic Mo Fractions and Isotopic Ratios in Sediments

To accurately estimate the Mo isotopic compositions of the authigenic components in each sample, it is essential to correct for the detrital Mo fractions present in deep-sea sediments. While both Aluminum (Al) and Titanium (Ti) are frequently used to estimate terrestrial material proportions and normalize detrital contributions when calculating authigenic fractions in both pelagic and terrigenous-dominated sediments[1, 2, 3], Ti offers a more precise representation of these terrestrial materials. Previous research indicates that a substantial amount of Al in marine sediments derives from non-terrestrial sources, including Al scavenged by Fe (hydro)oxide, biogenic opal, and clay minerals from seawater[1, 2, 3]. Supporting this, a sequential extraction procedure performed on the GC112 core in the present study revealed that the extracted non-terrestrial Al proportion (combining phosphate and Fe-Mn phases) varied between 10% and 30%, markedly exceeding that of Ti (1%-3%) (Supplementary Table S2). Moreover, in the GC112 core bulk sediments, the correlation between Ti and Zirconium (Zr)—also a conservative and stable element—(R^2 = 0.92) is more robust than that between Al and Zr (R^2 = 0.46). This disparity suggests Al in these sediments is more significantly influenced by authigenic components. Consequently, Ti is proposed for normalization due to its superior reliability as an indicator for quantifying detrital contributions in marine sediments. The authigenic fraction and isotopic ratio of sedimentary Mo can then be calculated using a mass balance method. The authigenic fraction and isotopic ratio of sedimentary Mo can be calculated using a mass balance method:

$$f_{Mo} = 1 – (Mo/ Ti)_{det} \times Ti_{bulk} / Mo_{bulk}$$

$$\delta^{98}Mo_{auth} = (\delta^{98}Mo_{bulk} – [(1 – f_{Mo}) \times \delta^{98}Mo_{det}]) / f_{Mo}$$

In these equations, Ti_{bulk} and Mo_{bulk} represent the Ti and Mo concentrations of the bulk samples, while $(Mo/Ti)_{det}$ is the elemental ratio of the detrital components, estimated using the

upper continental crust value (2.9 × 10^{-4})[4]. The $\delta^{98}Mo_{det}$ value is generally assumed to reflect the isotopic composition of detrital Mo (~0.30‰) [5, 6, 7, 8]. However, due to the extremely low abundance of Mo in the Earth's crust, the calculated Mo concentrations and isotopic compositions are consistent with the original values within the range of error in Supplementary Table S1. Consequently, the data presented in this study have not been adjusted for detrital Mo, except in the calculation of mean Mo content and isotopic composition in Deep-Sea Sediments.

S2. The results of Mo concentrations and isotope distributions in sequential extraction phases

A Mo elemental mass balance model was utilized to assess the effectiveness of the chemical extractions on the initial sequential extraction phase samples from GC112. The model is mathematically represented as:

$$[Mo]_{total} = \sum_{i=1}^{n}[Mo]i$$

where $[Mo]i$ denotes the concentration of Mo in phase i. In this analysis, i specifically refers to the phosphate, Mn oxide, Fe (hydro)oxide, and silicate phases. By accounting for the elemental contributions from each phase, the model provides a comprehensive evaluation of Mo partitioning. The calculated total Mo concentrations ($[Mo]$ total) are approximately 20% lower than the measured bulk Mo values (Supplementary Table S2). Given the strong positive correlation between the two datasets (R^2=0.95), it is hypothesized that this discrepancy may result from systematic instrumental errors or other unidentified factors. Major and trace element compositions for the three leachate phases are presented in Supplementary Table S2. For the second batch of Mo sequential extraction experiments on core sediments from GC112 and XT19, equilibrium calculations were not performed due to the lack of residual phase data. Nevertheless, the combined

Mo content in the extracted phases constitutes over 80% of the measured bulk Mo values in Supplementary Table S3. Isotopic mass balance calculations were not performed because Mo isotope data could not be obtained for several phases, as their Mo concentrations were too low to allow δ^{98}Mo measurement.

In the initial sequential extraction phase of the GC112 samples, Mo concentrations relative to the original sample weight were as follows: 0.7–3.5 μg g^{-1} in the phosphate phase (1.0%–10.3% of bulk sample Mo), 2.2–42.4 μg g^{-1} in the Mn oxide phase (2.4%–34.7% of bulk sample Mo), 7.2–47.3 μg g^{-1} in the Fe (hydro)oxide phase (29.5%–51.4% of bulk sample Mo), and 2.5–18.2 μg g^{-1} in the residue phase (7.4%–19.9% of bulk sample Mo) (Supplementary Table S2). For the second batch of Mo sequential extraction experiments, results varied by core (Supplementary Table S3). In the GC112 core sediments, Mo concentrations were 3.8–21.4 μg g^{-1} in the phosphate phase (3.7%–34.4% of bulk sample Mo) and 7.6–70.9 μg g^{-1} in the Fe-Mn (hydro)oxide phase (44.2%–61.5% of bulk sample Mo). In the XT19 core sediments, Mo concentrations measured 0.96–1.45 μg g^{-1} in the phosphate phase (2.82%–5.49% of bulk sample Mo) and 18.0–36.2 μg g^{-1} in the Fe-Mn (hydro)oxide phase (84.6%–92.4% of bulk sample Mo). Regarding isotopic composition, the δ^{98}Mo values in the Fe-Mn (hydro)oxide phase of the GC112 samples ranged from −0.86‰ to 0.04‰ (Supplementary Table S3), indicating a slight depletion compared to bulk rock values (Fig. 2). Conversely, the δ^{98}Mo signatures in the Fe-Mn (hydro)oxide phase of XT19 samples (from −0.47‰ to 0.04‰) closely approximated those of their corresponding bulk samples (Fig. 2). Notably, despite these minor differences, the isotopic variation patterns in the Fe-Mn (hydro)oxide phase of both cores broadly mirrored those exhibited by the bulk samples.

S3. Calculation of Mean Mo Content and Isotopic Composition in Deep-Sea Sediments

Mo content data, comprising over 2,000 depth measurements from more than 80 sediment cores collected across the Pacific, Indian, and Atlantic Oceans, were analyzed [8, 9, 10, 11, 12, 13, 14.] Mo isotope data were obtained from both previous studies [10, 11] and our investigation. Due to the heterogeneous physical and chemical properties of deep-sea sediments, Mo content and isotopic composition exhibit significant variability with depth [8, 9, 10, 11, 12, 13, 14]. To accurately estimate the mean Mo content and isotopic composition in deep-sea sediments, a high-resolution depth-averaging method was applied. A 0.5-meter depth interval, based on the greatest common divisor of core sampling intervals, was used for calculating average Mo content. In sections between 50 and 70 meters, where some sampling intervals exceeded 0.5 meters, a 1.0-meter resolution facilitated statistically robust mean calculations. The Mo content and isotopic composition of these intervals were then averaged to represent the overall marine sediment composition (Fig. S6). This method, commonly employed to determine the geochemical composition of global riverine sediments and soils[15, 16], provides a robust estimation of average values. Results indicate a mean Mo content of 46.0±30 ppm across the entire deep-sea pelagic sediment layer (approximately 73 meters deep) and a mean isotopic composition of –0.08‰ (approximately 7.5 meters deep). Furthermore, the average authigenic Mo content and isotopic composition were calculated as 44.9 ±30 ppm and –0.09± 0.23‰, respectively, using the procedure detailed in Section S1.

S4. Monte Carlo simulations of the oceanic Mo isotopes mass balance simulation

The Mo fluxes and isotopic compositions within reducing and euxinic settings can be calculated with the mass balance simulation:

$$F_{RIV} + F_{L\text{-}hyd} = F_{OX} + F_{RED} + F_{EUX} + F_{H\text{-}hyd} \quad (1)$$

$$F_{RIV} \times \delta^{98}Mo_{RIV} + F_{L\text{-}hyd} \times \delta^{98}Mo_{L\text{-}hyd} = F_{OX} \times \delta^{98}Mo_{OX} + F_{RED} \times \delta^{98}Mo_{RED} + F_{EUX} \times \delta^{98}Mo_{EUX} + F_{H\text{-}hyd} \times \delta^{98}Mo_{H\text{-}hyd} \quad (2)$$

Where F terms represent Mo fluxes (10^8 mol/yr) from various environments: Riverine (F_{RIV}), Low-temperature hydrothermal ($F_{L\text{-}hyd}$), High-temperature hydrothermal ($F_{H\text{-}hyd}$), Oxic (F_{OX}), Reducing (F_{RED}), and Euxinic (F_{EUX}) sedimentary environments. The corresponding δ^{98}Mo terms denote Mo isotope compositions for each source or sink, with values and ranges presented in Supplementary Table S3.

The parameters used in this study include F_{RIV} (3.1×10^8 mol yr^{-1}), $F_{L\text{-}hyd}$ (0.26×10^8 mol yr^{-1}), F_{OX} (1.52×10^8 mol yr^{-1}), $F_{H\text{-}hyd}$ (0.1×10^8 mol yr^{-1}), $\delta^{98}Mo_{RIV}$ (0.80‰), $\delta^{98}Mo_{L\text{-}hyd}$ (0.80‰), $\delta^{98}Mo_{OX}$ (–0.09‰), and $\delta^{98}Mo_{H\text{-}hyd}$ (–0.5‰) in the Supplementary Table S4. A Monte Carlo simulation with 100,000 iterations was performed, constraining F_{RED} to a range of 0.8 to 1.65 × 10^8 mol yr^{-1}, F_{EUX} to 0.198 to 0.495 × 10^8 mol yr^{-1}, $\delta^{98}Mo_{SAD}$ to –0.09‰ to 1.8‰, and $\delta^{98}Mo_{EUX}$ to 1.8‰ to 2.3‰. Analysis of the equilibrium calculations indicates a moderate negative correlation between the total error and both F_{RED} ($R^2 = 0.15$, $p<0.001$) and $\delta^{98}Mo_{RED}$ ($R^2 = 0.69$, $p<0.001$). The total error is defined as the absolute sum of the errors in the Mo mass balance and isotopic balance. In contrast, neither F_{EUX} nor $\delta^{98}Mo_{EUX}$ shows a significant linear relationship with the total error. These findings suggest that variations in F_{RED} and $\delta^{98}Mo_{RED}$ are the primary contributors to the total error in the equilibrium calculation, while F_{EUX} and $\delta^{98}Mo_{EUX}$ have a negligible impact. The optimal parameter set, identified by minimizing the total error (the sum of Mo mass balance and isotopic balance errors), achieves a minimum total error of 0.004066 over 100,000 iterations. These optimal values are $F_{RED} = 1.45 \times 10^8$ mol yr^{-1}, $F_{EUX} = 0.28 \times 10^8$ mol yr^{-1}, $\delta^{98}Mo_{RED} = 1.61$‰, and $\delta^{98}Mo_{EUX} = 1.90$‰, respectively (Fig. S7 and Supplementary Table S4).

S5. Mass balance model of Mo cycle

Assuming a steady-state marine molybdenum (Mo) cycle, the $\delta^{98}Mo$ signature of global seawater predominantly reflects the partitioning of Mo among sediments deposited under three prevailing redox conditions: euxinic, anoxic (or suboxic), and oxic environments[17, 18]. When riverine input is regarded as the principal source of oceanic Mo, its flux is typically standardized to modern values for consistency. Under these conditions, the isotopic composition of seawater Mo is determined by the relative fluxes to each redox sink, as described by the relationship:

$$F_{EUX} + F_{RED} + F_{OX} = F_{Rivers}$$

The isotopic mass balance is expressed as:

$$\delta^{98}Mo_{river} = F_{OX} \times (\delta^{98}Mo_{SW} + \Delta_{OX}) + F_{RED} \times (\delta^{98}Mo_{SW} + \Delta_{RED}) + F_{EUX} \times (\delta^{98}Mo_{SW} + \Delta_{EUX})$$

where F_{OX}, F_{RED}, and F_{EUX} represent Mo output flux to each sediment type. $\delta^{98}Mo_{river}$ and $\delta^{98}Mo_{SW}$ denote the isotopic compositions of the riverine input and seawater, respectively, while Δ refers to the isotopic fractionation between seawater and each sedimentary flux (*OX* = oxic sink; *RED* = reducing sink; *EUX* = euxinic sink). The necessary parameters, including the Mo fluxes for sediment output and riverine input, as well as fractionation magnitudes, are provided for the modern ocean (Supplementary Table S4). It is worth emphasizing that $\delta^{98}Mo_{river}$ is provisionally set at 0.7‰[19]. To obtain $\delta^{98}Mo_{SW}$ as a function of the removal fluxes (F_{OX}, F_{RED}, and F_{EUX}), we solve the equations above and subsequently plot the contours of $\delta^{98}Mo_{SW}$ in Fig. 5.

FIGURES

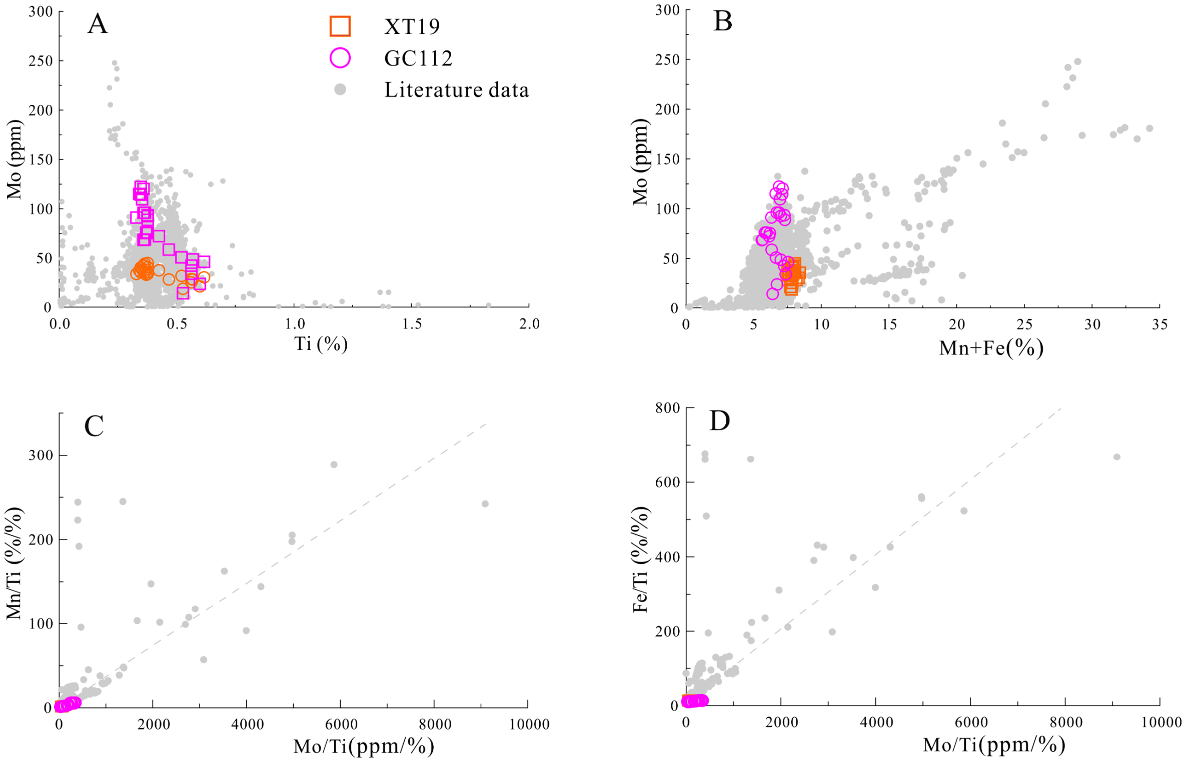

Fig. S1. Geochemical relationships of Mo in deep-sea sediments from the Indian and Pacific Oceans. The panels show relationships between: (A) Mo vs. Ti concentrations, (B) Mo vs. (Mn+Fe) concentrations, (C) Mo/Ti vs. Mn/Ti ratios, and (D) Mo/Ti vs. Fe/Ti ratios. All data are from bulk-sediment samples (n=1955). The complete dataset is available in the Supplementary Dataset 1-2 hosted on Mendeley Data[20].

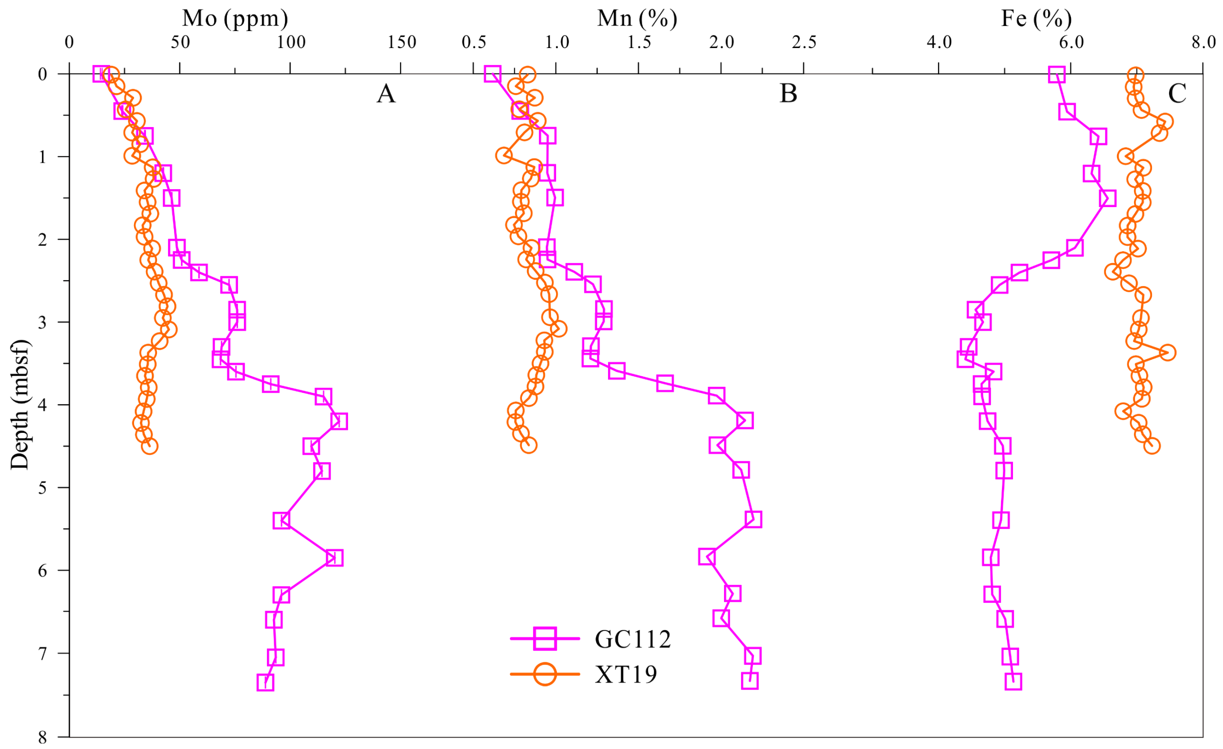

Fig. S2. Downcore variations of Mo, Mn, and Fe in western Pacific deep-sea sediments. The plots show concentration profiles for Mo, Mn, and Fe in cores GC112 and XT19. Depth is given in meters below seafloor (mbsf).

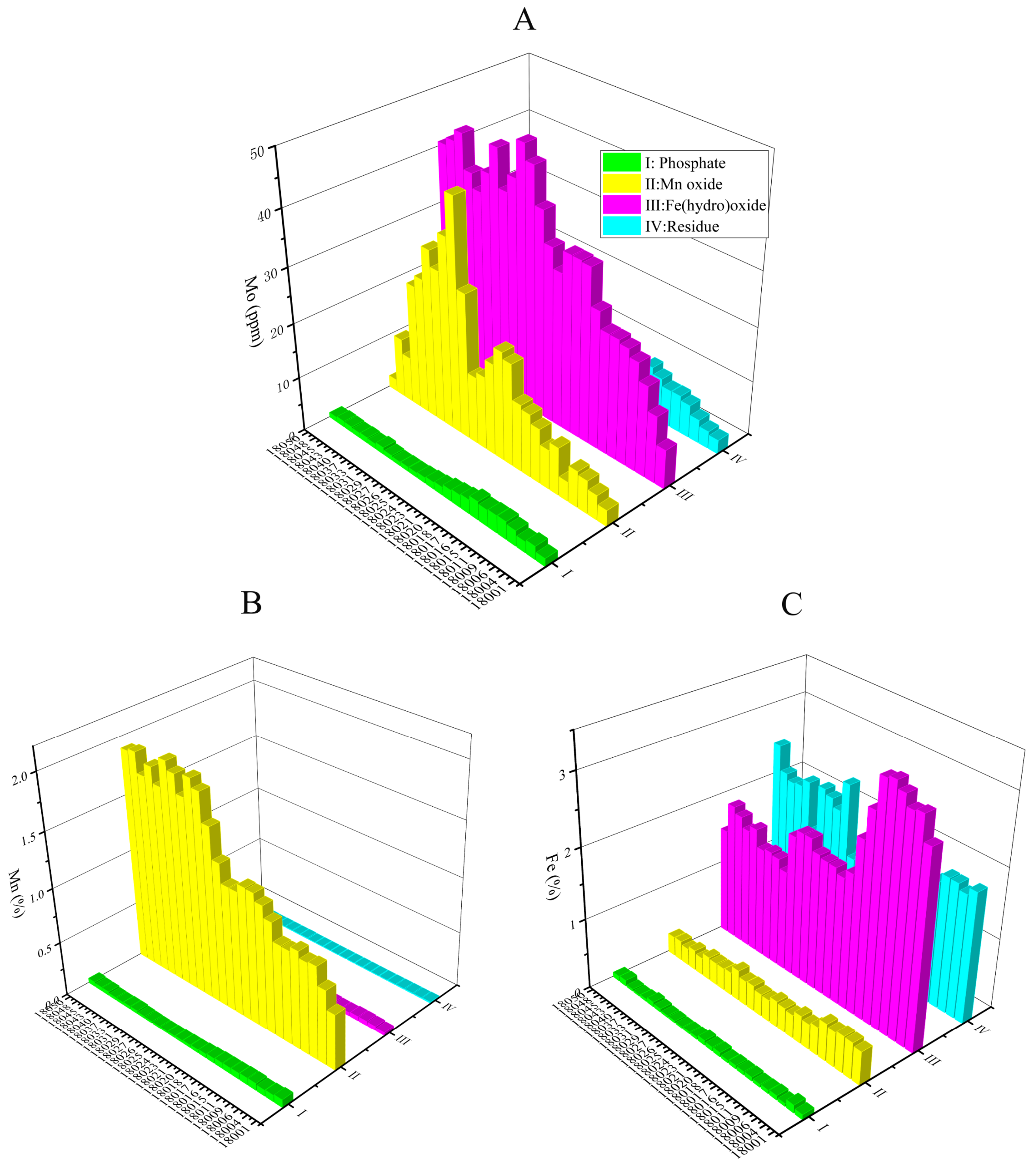

Fig. S3. Molybdenum, Mn, and Fe partitioning in sediment core GC112. The bar charts show the concentrations of (A) Mo, (B) Mn, and (C) Fe in four operationally defined geochemical phases, as determined by sequential extraction. The phases are: (I) phosphate-bound, (II) Mn oxides, (III) Fe (hydro)oxides, and (IV) residual. All concentrations are reported relative to the initial bulk-sediment weight.

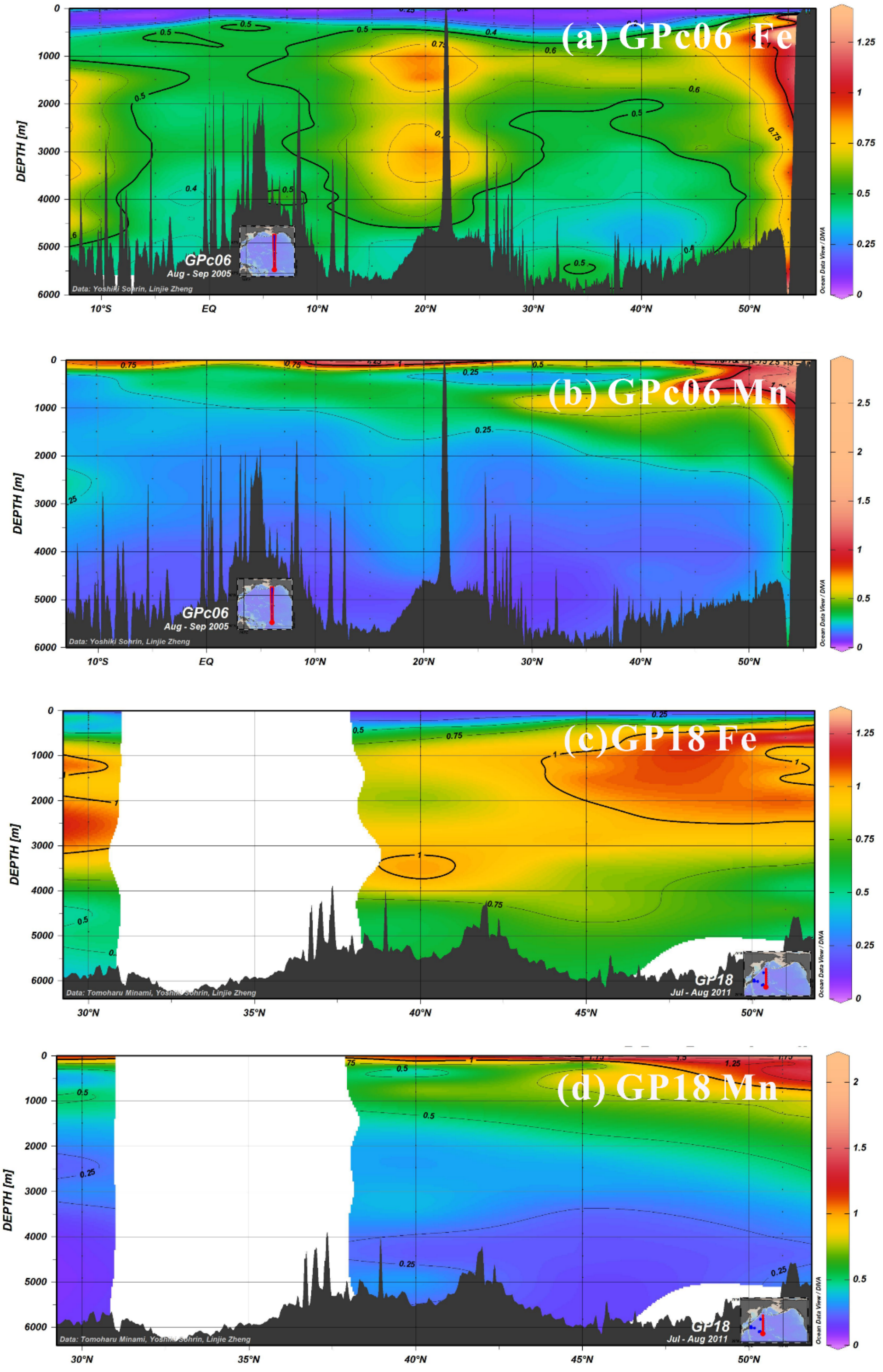

Fig. S4. Dissolved Fe and Mn along North Pacific GEOTRACES transects. Section plots showing dissolved Fe and Mn concentrations from cruises GP18 and GPc06. Data are from the eGEOTRACES Electronic Atlas[21].

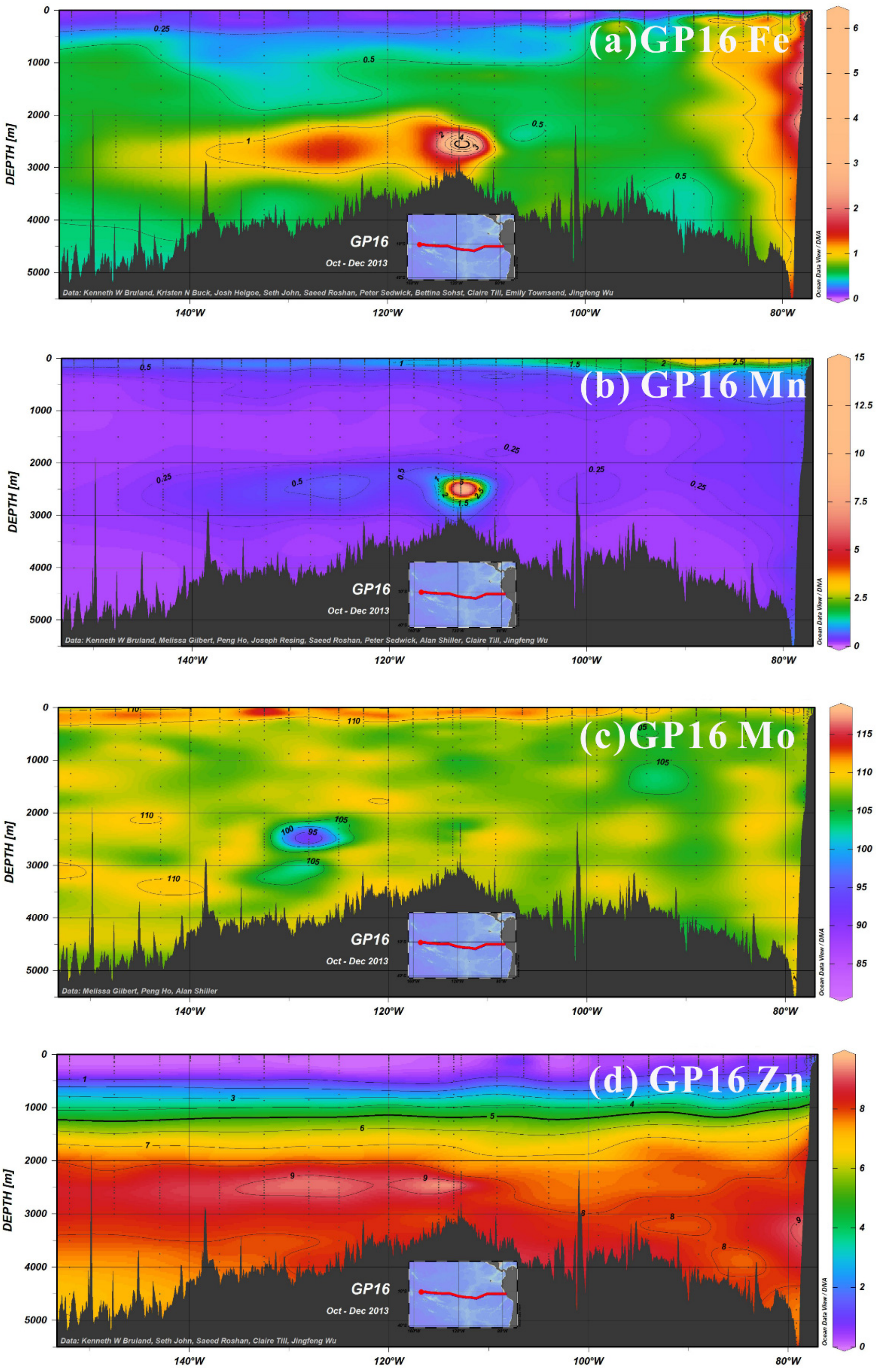

Fig. S5. Dissolved trace metal concentrations along the Eastern Pacific GEOTRACES transect. The section plots show dissolved Fe, Mn, Mo, and Zn along cruise GP16. Data are from the eGEOTRACES Electronic Atlas[21].

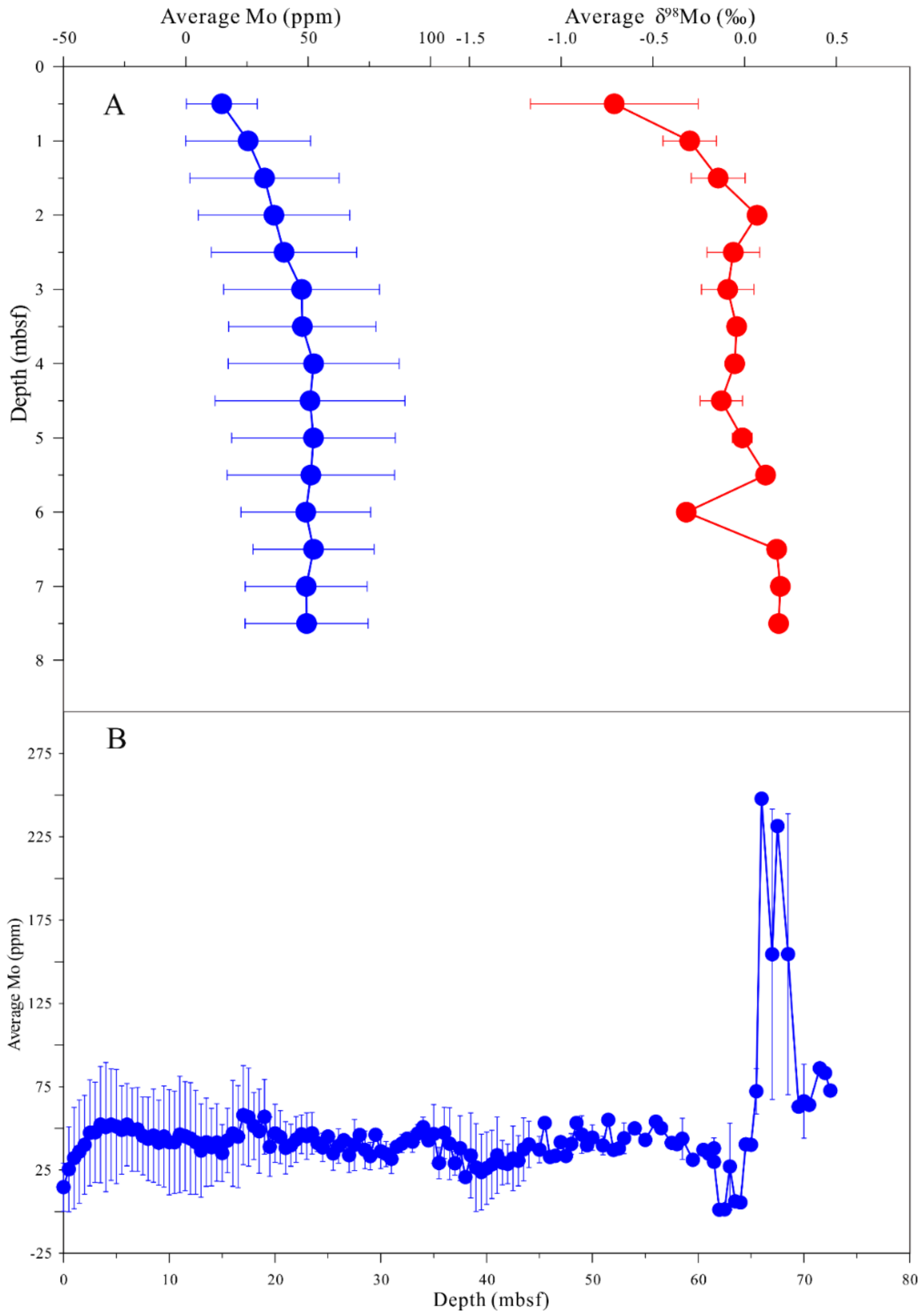

Fig. S6. Mean Mo isotope composition and content versus depth in global pelagic deep-sea sediments. (A) The mean Mo isotope composition is plotted against depth (0–7.5 m, at a 0.5 m resolution). (B) The mean Mo content is plotted against depth (0–73 m, at a 0.5 m resolution). The complete dataset is available in the Supplementary Dataset 1-2 hosted on Mendeley Data[20].

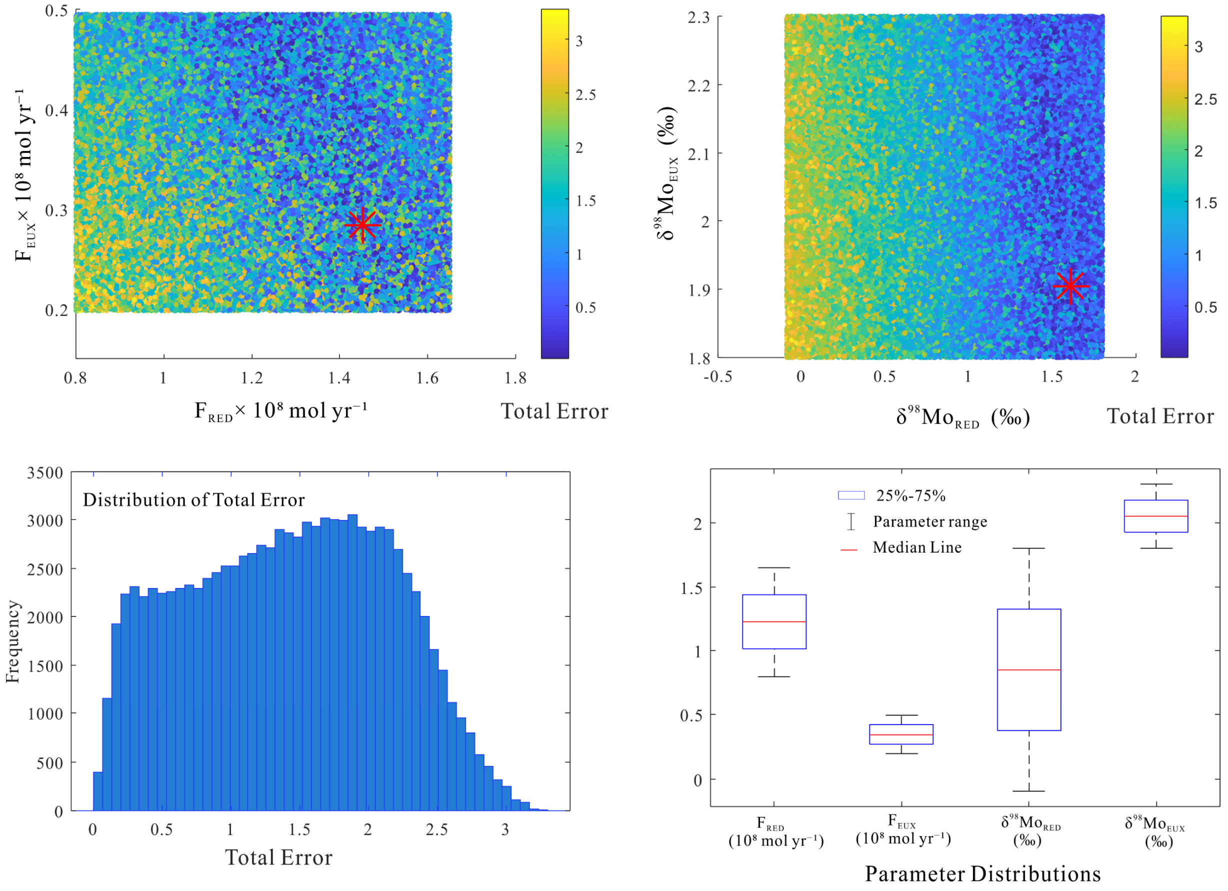

Fig. S7. Sensitivity analysis of the Mo isotope mass balance model. Results from 10,000 Monte Carlo simulations testing the model's sensitivity to input parameters, which are defined in Supplementary Table S4. Each point represents a single model run. The star indicates the optimal solution that best fits modern seawater values.

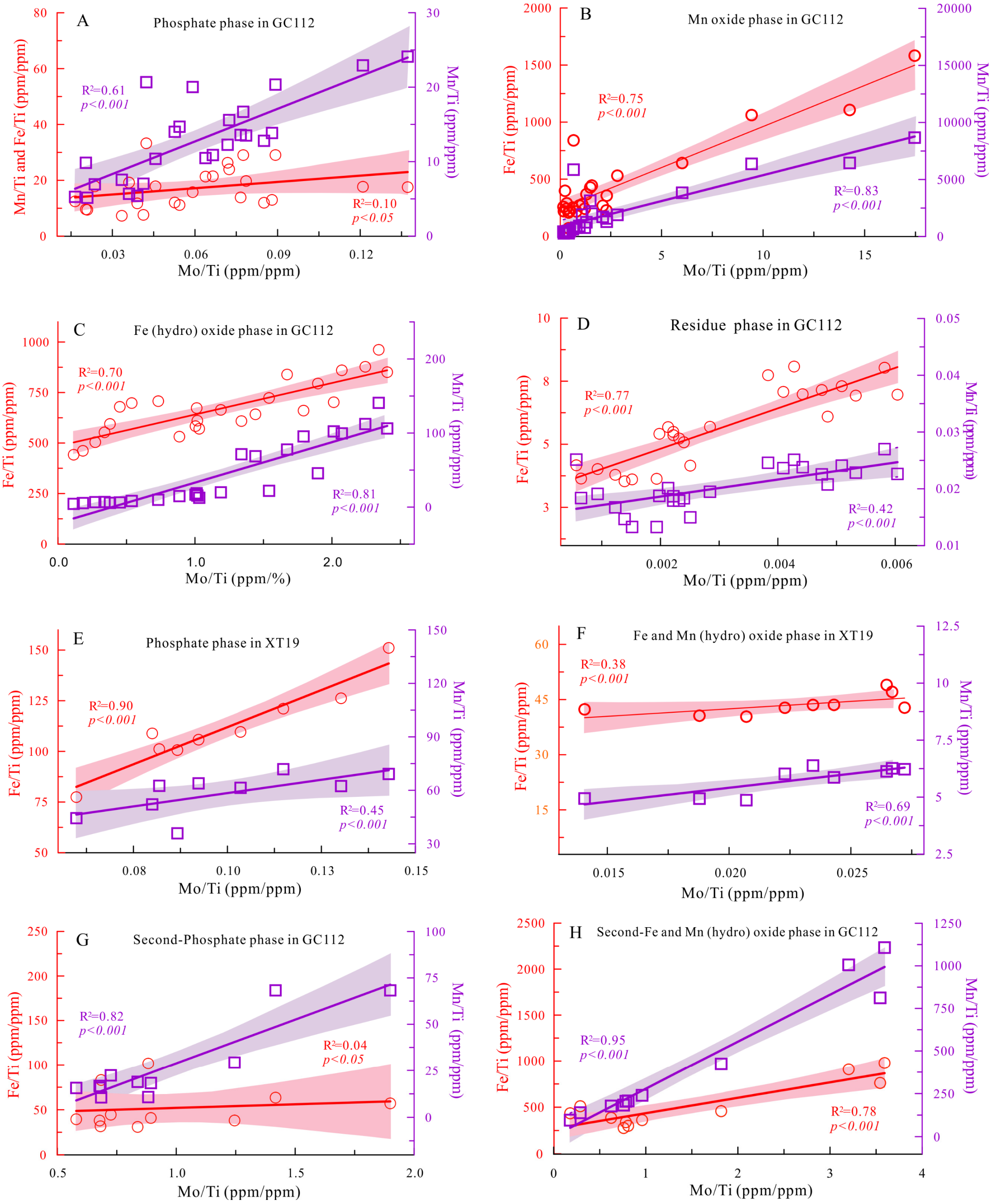

Fig. S8. Covariation of Mo/Ti with Mn/Ti and Fe/Ti ratios in sequentially extracted sediment phases. Mo/Ti ratios demonstrated a linear correlation with both Mn/Ti and Fe/Ti ratios. This was observed in four phases (phosphate (A), Mn oxides (B), Fe (hydro)oxide (C), and residual phase (D)) sequentially extracted

from the GC112 sample, and in two phases (phosphate and Fe-Mn (hydro)oxide) similarly extracted from the XT19 (E and F) and GC112 (G and H) core samples. The shaded area represents the 95% confidence interval. Fe in the deep-sea sediments of cores XT19 and GC112, which is present predominantly as (hydro)oxides, originates mainly from atmospheric dust and volcanic ash. Consequently, normalizing Fe concentrations to Ti across the various extraction phases is essential for isolating the authigenic Fe signal.

Supplementary Table S1. Mo isotopes and selected major and trace element compositions of the deep-sea sediments in Western Picific Ocean. Fe, Mn, and Al contents in GC112 core from previous studies[22, 23]

Sample	Depth	Mn	Fe	Al	Ti	Bulk Mo	Mo_{auth}	Co	Mn/Ti	Fe/Ti	Mo/Ti	Mn/Mo	Fe/Mo	Co/Mn	Bulk δ^{98}Mo	$\delta^{98}Mo_{auth}$	2SE
	mbsf	%	%	%	%	ppm	ppm	ppm	%/%	%/%	ppm/%	ppm/ppm	%/ppm	ppm/%	‰	‰	
**GC112**																	
18001	0.00	0.62	5.79	8.87	0.53	14.5	13.3	103	1.17	11.0	27.4	427	0.40	167	-0.48	-0.55	0.04
18004	0.45	0.78	5.95	8.88	0.60	24.0	22.8	119	1.31	9.9	40.0	327	0.25	152	-0.55	-0.59	0.04
18006	0.75	0.95	6.42	8.84	0.56	34.2	33.1	147	1.69	11.4	60.7	278	0.19	155	-0.54	-0.57	0.03
18009	1.20	0.95	6.32	8.90	0.56	42.5	41.4	151	1.68	11.2	75.5	223	0.15	159	-0.39	-0.41	0.02
18011	1.50	1.00	6.56	8.69	0.62	46.3	45.2	156	1.61	10.6	75.0	215	0.14	157	-0.24	-0.25	0.03
18015	2.10	0.95	6.06	8.86	0.57	48.7	47.6	158	1.66	10.6	85.6	194	0.12	167	-0.17	-0.18	0.02
18016	2.25	0.95	5.71	8.61	0.52	50.9	49.8	152	1.82	10.9	97.6	187	0.11	160	-0.14	-0.15	0.02
18017	2.40	1.11	5.22	7.98	0.47	58.7	57.7	167	2.38	11.2	126	189	0.09	150	-0.28	-0.29	0.04
18018	2.55	1.22	4.92	7.70	0.43	72.4	71.4	175	2.88	11.6	170	169	0.07	143	-0.32	-0.32	0.03
18020	2.85	1.29	4.56	7.68	0.37	76.0	75.0	184	3.47	12.3	204	170	0.06	142	-0.26	-0.27	0.02
18021	3.00	1.29	4.67	7.37	0.38	76.0	75.1	172	3.42	12.4	201	170	0.06	133	-0.17	-0.18	0.04
18023	3.30	1.21	4.46	7.29	0.37	68.9	68.0	162	3.31	12.2	188	176	0.06	134	-0.06	-0.06	0.04
18024	3.45	1.21	4.41	7.19	0.36	68.5	67.5	160	3.36	12.3	190	177	0.06	132	-0.04	-0.05	0.05
18025	3.60	1.37	4.83	7.35	0.37	75.5	74.5	175	3.68	13.0	203	181	0.06	128	-0.06	-0.07	0.05
18026	3.75	1.66	4.65	7.49	0.33	91.2	90.2	205	5.04	14.1	277	182	0.05	123	-0.08	-0.09	0.03
18027	3.90	1.97	4.66	8.26	0.34	115	114.1	243	5.78	13.6	337	171	0.04	123	-0.04	-0.05	0.03
18029	4.20	2.15	4.74	8.16	0.35	122	121.2	263	6.17	13.6	351	176	0.04	123	-0.31	-0.32	0.04
18031	4.50	1.98	4.97	8.41	0.35	110	108.5	243	5.59	14.1	310	181	0.05	123	0.01	0.00	0.02
18033	4.80	2.12	4.99	8.13	0.35	114	113.4	271	6.10	14.4	329	185	0.04	128	0.04	0.04	0.03
18037	5.40	2.20	4.94	8.12	0.36	96.1	95.0	273	6.11	13.7	267	229	0.05	124	0.11	0.11	0.03
18040	5.85	1.91	4.79	8.17	0.37	120	119.2	228	5.24	13.1	329	159	0.04	119	-0.32	-0.32	0.02
18043	6.30	2.07	4.81	7.83	0.36	96.0	95.0	256	5.76	13.4	267	216	0.05	124	0.17	0.17	0.02
18045	6.60	2.00	5.01	7.90	0.38	92.6	91.6	256	5.30	13.3	245	216	0.05	128	0.19	0.19	0.05
18048	7.05	2.19	5.08	7.71	0.38	93.5	92.5	268	5.81	13.5	248	235	0.05	122	0.19	0.19	0.02
18050	7.35	2.17	5.13	7.62	0.38	88.8	87.8	267	5.76	13.6	235	245	0.06	123	0.18	0.18	0.02
**TX19**																	
XT19-1	0.01	0.83	6.98	10.17	0.51	19.0	17.7	78	1.63	13.7	37.2	436	0.37	94	-0.52	-0.58	0.04
XT19-8	0.15	0.76	6.95	10.31	0.52	21.4	20.1	71	1.45	13.3	40.8	354	0.32	94	-0.46	-0.51	0.04
XT19-15	0.29	0.87	6.98	10.25	0.51	28.8	27.5	80	1.71	13.6	56.4	303	0.24	92	-0.41	-0.45	0.03
XT19-22	0.43	0.78	7.07	10.64	0.53	25.5	24.1	72	1.47	13.4	48.2	305	0.28	93	-0.44	-0.48	0.04
XT19-29	0.57	0.89	7.43	10.89	0.54	30.6	29.2	78	1.65	13.8	56.8	291	0.24	87	-0.38	-0.41	0.04
XT19-36	0.71	0.81	7.35	10.80	0.53	28.5	27.1	72	1.53	13.9	53.9	285	0.26	89	-0.24	-0.26	0.05
XT19-43	0.85					32.1									-0.23		0.03
XT19-50	0.99	0.69	6.83	10.10	0.50	28.5	27.2	64	1.36	13.6	56.7	241	0.24	94	-0.12	-0.14	0.03
XT19-57	1.13	0.87	7.10	10.11	0.50	37.7	36.4	80	1.73	14.1	74.9	230	0.19	92	-0.02	-0.04	0.03
XT19-64	1.27	0.85	6.98	9.92	0.49	38.2	36.9	74	1.72	14.2	77.5	222	0.18	87	-0.03	-0.05	0.04

XT19-71	1.41	0.79	7.09	10.21	0.51	34.1	32.7	69	1.54	13.8	66.5	232	0.21	87	-0.04	-0.05	0.03
XT19-78	1.55	0.79	7.09	10.23	0.51	35.4	34.1	66	1.55	13.9	69.7	222	0.20	84	0.03	0.02	0.04
XT19-85	1.69	0.81	6.98	10.13	0.50	36.7	35.4	68	1.63	14.1	74.0	220	0.19	85	0.12	0.11	0.08
XT19-92	1.83	0.75	6.86	10.20	0.51	33.2	31.9	63	1.48	13.6	65.7	225	0.21	84	0.03	0.01	0.04
XT19-99	1.97	0.77	6.86	10.19	0.51	34.1	32.8	66	1.52	13.5	67.0	227	0.20	85	0.09	0.08	0.04
XT19-106	2.11	0.85	7.02	10.19	0.51	37.5	36.2	73	1.68	13.8	73.9	227	0.19	85	0.07	0.06	0.04
XT19-113	2.25	0.82	6.79	9.93	0.50	35.7	34.4	73	1.63	13.5	71.2	230	0.19	89	0.07	0.06	0.04
XT19-120	2.39	0.88	6.64	9.64	0.50	38.6	37.4	80	1.77	13.4	77.9	227	0.17	91	0.09	0.08	0.05
XT19-127	2.53	0.93	6.88	9.80	0.50	40.5	39.2	81	1.85	13.6	80.1	231	0.17	86	0.02	0.01	0.04
XT19-134	2.67	0.96	7.10	9.94	0.51	42.9	41.6	83	1.89	14.0	84.4	224	0.17	87	0.02	0.01	0.04
XT19-141	2.81					44.5									0.05		0.03
XT19-148	2.95	0.96	7.06	9.84	0.51	42.3	41.1	85	1.91	14.0	83.8	228	0.17	88	0.01	0.00	0.04
XT19-155	3.09	1.02	7.03	9.67	0.50	45.1	43.8	88	2.03	14.0	89.8	226	0.16	86	-0.01	-0.01	0.03
XT19-162	3.23	0.93	6.96	9.85	0.51	40.9	39.6	85	1.83	13.7	80.4	227	0.17	91	-0.02	-0.03	0.04
XT19-169	3.37	0.93	7.47	10.39	0.53	35.7	34.4	87	1.76	14.1	67.5	261	0.21	93	-0.09	-0.11	0.04
XT19-176	3.51	0.91	6.99	9.81	0.50	35.5	34.2	88	1.82	14.0	71.3	255	0.20	97	-0.04	-0.06	0.03
XT19-183	3.65	0.88	7.04	9.90	0.50	34.3	33.0	88	1.77	14.2	68.9	257	0.21	99	-0.02	-0.03	0.04
XT19-190	3.79	0.88	7.10	9.76	0.48	36.0	34.7	84	1.81	14.7	74.4	244	0.20	95	-0.03	-0.05	0.04
XT19-197	3.93	0.84	7.08	9.84	0.49	35.1	33.8	87	1.71	14.5	71.8	239	0.20	104	-0.10	-0.11	0.04
XT19-204	4.08	0.76	6.79	9.41	0.48	33.6	32.4	84	1.59	14.3	70.5	225	0.20	110	0.00	-0.01	0.04
XT19-211	4.22	0.76	7.03	9.74	0.50	32.4	31.2	83	1.52	14.2	65.4	233	0.22	110	-0.09	-0.11	0.03
XT19-218	4.36	0.79	7.09	9.77	0.50	33.8	32.5	86	1.56	14.1	67.0	233	0.21	109	-0.10	-0.12	0.04
XT19-225	4.50	0.84	7.23	9.80	0.50	36.4	35.1	89	1.66	14.3	72.2	230	0.20	106	-0.09	-0.10	0.03

Supplementary Table S2. Molybdenum, Mn, Fe, Ti, and Al concentration in bulk sample and four sequential extractions data of the GC112 core.

**Mo**	**Bulk sample**	**Phosphate phase**		**Mn oxide phase**		**Fe (hydro) oxide phase**		**Residue phase**		**Mn**	**Bulk sample**	**Phosphate phase**		**Mn oxide phase**		**Fe (hydro) oxide phase**		**Residue phase**	
Sample ID	Mo	Mo[1]	Ratios[2]	Mo	Ratios	Mo	Ratios	Mo	Ratios	Sample ID	Mn	Mn	Ratios	Mn	Ratios	Mn	Ratios	Mn	Ratios
	μg/g	μg/g	%	μg/g	%	μg/g	%	μg/g	%		%								
18001	14.5	1.5	10.3	3.0	20.4	7.2	49.8	2.5	17.5	18001	0.62	0.07	11.61	0.51	82	0.03	4.49	0.01	1.82
18004	24.0	2.5	10.3	4.5	18.9	12.3	51.4	3.1	13.1	18004	0.78	0.06	7.77	0.68	86	0.04	4.70	0.01	1.14
18006	34.2	2.0	6.0	6.1	17.8	16.7	48.9	4.3	12.5	18006	0.95	0.06	5.81	0.85	89	0.04	4.33	0.01	0.93
18009	42.5	2.9	6.8	6.7	15.7	20.0	47.1	6.0	14.2	18009	0.95	0.07	6.87	0.83	88	0.04	4.31	0.01	0.87
18011	46.3	3.3	7.2	3.6	7.7	21.5	46.3	6.8	14.8	18011	1.00	0.06	6.37	0.89	90	0.03	3.27	0.01	0.73
18015	48.7	3.3	6.7	8.8	18.0	22.1	45.4	6.8	13.9	18015	0.95	0.06	6.09	0.85	90	0.03	3.29	0.01	0.63
18016	50.9	3.1	6.1	7.2	14.2	22.2	43.5	8.1	16.0	18016	0.95	0.05	5.77	0.86	90	0.03	3.60	0.01	0.59
18017	58.7	3.5	5.9	10.1	17.1	25.3	43.0	9.3	15.9	18017	1.11	0.05	4.71	1.02	92	0.03	3.06	0.01	0.50

18018	72.4	2.6	3.7	11.7	16.1	32.0	44.3	7.5	10.3	18018	1.22	0.05	3.69	1.12	92	0.05	4.02	0.01	0.47
18020	76.0	2.4	3.1	12.4	16.3	32.3	42.5	7.0	9.3	18020	1.29	0.04	2.89	1.19	92	0.05	4.20	0.01	0.42
18021	76.0	2.1	2.8	18.8	24.7	32.4	42.6	7.7	10.1	18021	1.29	0.03	2.25	1.20	93	0.05	4.21	0.01	0.41
18023	68.9	1.5	2.2	19.9	28.9	28.4	41.2	5.7	8.3	18023	1.21	0.03	2.15	1.13	93	0.05	4.32	0.00	0.38
18024	68.5	1.3	1.9	16.8	24.5	32.1	46.8	5.3	7.7	18024	1.21	0.02	1.71	1.13	93	0.05	4.44	0.01	0.41
18025	75.5	1.0	1.4	13.5	17.8	37.7	49.9	5.5	7.3	18025	1.37	0.02	1.25	1.30	95	0.05	3.36	0.01	0.38
18026	91.2	0.9	1.0	12.4	13.6	44.4	48.7	5.8	6.3	18026	1.66	0.01	0.85	1.58	95	0.06	3.82	0.00	0.29
18027	115.1	0.8	0.7	26.6	23.1	47.3	41.1	16.7	14.5	18027	1.97	0.01	0.73	1.84	93	0.11	5.77	0.01	0.40
18029	122.2	0.7	0.6	42.4	34.7	40.7	33.3	13.1	10.7	18029	2.15	0.02	0.76	1.92	89	0.21	9.64	0.01	0.29
18031	109.6	0.9	0.8	34.7	31.7	37.6	34.4	10.4	9.5	18031	1.98	0.02	0.99	1.73	87	0.23	11.44	0.01	0.34
18033	114.4	1.3	1.1	28.0	24.4	44.5	38.9	11.4	10.0	18033	2.12	0.03	1.33	1.89	89	0.20	9.26	0.01	0.32
18037	96.1	0.9	1.0	30.8	32.1	39.4	41.0	11.9	12.3	18037	2.20	0.03	1.24	1.97	90	0.20	8.96	0.01	0.23
18040	120.2	1.1	0.9	24.8	20.6	35.4	29.5	12.4	10.3	18040	1.91	0.02	1.19	1.72	90	0.16	8.60	0.01	0.37
18043	96.0	0.9	1.0	22.6	23.6	38.0	39.6	12.9	13.4	18043	2.07	0.02	1.18	1.86	90	0.18	8.74	0.01	0.34
18045	92.6	1.1	1.1	8.3	9.0	44.2	47.7	15.5	16.7	18045	2.00	0.04	1.78	1.75	87	0.21	10.60	0.01	0.33
18048	93.5	1.1	1.2	10.9	11.7	42.2	45.1	18.2	19.5	18048	2.19	0.04	1.62	1.93	88	0.22	10.25	0.01	0.31
18050	88.8	0.9	1.0	2.2	2.4	40.8	46.0	17.7	19.9	18050	2.17	0.04	2.00	1.91	88	0.22	9.99	0.01	0.38

**Fe**	Fe	Fe	Ratios	Fe	Ratios	Fe	Ratios	Fe	Ratios	**Ti**	Ti	Ti	Ratios	Ti	Ratios	Ti	Ratios	Ti	Ratios
Sample ID	%									Sample ID	µg/g	µg/g	%	µg/g	%	µg/g	%	µg/g	%
18001	5.79	0.07	1.21	0.48	8.21	2.79	48.16	1.87	32.23	18001	5276	72.8	1.38	11.96	0.23	63.14	1.20	4482	84.95
18004	5.95	0.11	1.88	0.52	8.70	3.13	52.67	1.77	29.75	18004	5995	118.3	1.97	23.66	0.39	67.91	1.13	4861	81.08
18006	6.42	0.04	0.65	0.52	8.16	3.10	48.34	1.85	28.85	18006	5635	37.6	0.67	18.20	0.32	61.60	1.09	4615	81.89
18009	6.32	0.06	1.00	0.50	7.86	3.26	51.57	1.86	29.44	18009	5635	86.1	1.53	20.64	0.37	58.96	1.05	4909	87.11
18011	6.56	0.05	0.75	0.53	8.10	3.36	51.25	1.74	26.53	18011	6175	27.7	0.45	20.72	0.34	56.59	0.92	4929	79.82
18015	6.06	0.04	0.69	0.40	6.57	3.33	54.87	1.61	26.63	18015	5695	23.9	0.42	18.00	0.32	49.02	0.86	4477	78.61
18016	5.71	0.06	0.98	0.36	6.25	2.87	50.34	1.52	26.70	18016	5216	40.3	0.77	17.20	0.33	41.20	0.79	4199	80.51
18017	5.22	0.05	0.94	0.40	7.63	2.44	46.69	1.54	29.57	18017	4676	40.9	0.87	15.91	0.34	34.51	0.74	3722	79.59
18018	4.92	0.05	0.99	0.33	6.67	1.92	39.04	1.68	34.21	18018	4256	64.2	1.51	12.22	0.29	31.50	0.74	3224	75.74
18020	4.56	0.03	0.77	0.35	7.66	1.80	39.55	1.49	32.65	18020	3717	26.9	0.72	9.51	0.26	27.18	0.73	2933	78.90
18021	4.67	0.06	1.35	0.37	7.93	1.88	40.24	1.54	32.91	18021	3777	53.6	1.42	15.40	0.41	32.25	0.85	2703	71.58
18023	4.46	0.06	1.26	0.31	6.90	1.89	42.36	1.41	31.69	18023	3657	21.3	0.58	8.66	0.24	28.06	0.77	2573	70.35
18024	4.41	0.07	1.59	0.31	7.14	1.92	43.62	1.45	32.84	18024	3597	36.4	1.01	5.93	0.16	36.19	1.01	2674	74.35

18025	4.83	0.03	0.72	0.36	7.52	2.08	43.10	1.46	30.23	18025	3717	16.4	0.44	16.74	0.45	36.54	0.98	2574	69.25
18026	4.65	0.03	0.60	0.35	7.51	2.08	44.78	1.40	30.06	18026	3297	13.0	0.40	7.88	0.24	28.82	0.87	2610	79.14
18027	4.66	0.02	0.45	0.43	9.30	1.98	42.47	2.39	51.32	18027	3417	10.6	0.31	62.39	1.83	24.92	0.73	3276	95.86
18029	4.74	0.03	0.59	0.33	6.93	1.42	29.92	1.97	41.57	18029	3477	15.7	0.45	2.97	0.09	20.23	0.58	2759	79.34
18031	4.97	0.03	0.56	0.31	6.33	1.54	31.06	2.09	42.03	18031	3537	9.6	0.27	1.99	0.06	16.08	0.45	2703	76.42
18033	4.99	0.05	0.98	0.31	6.30	1.57	31.49	2.16	43.25	18033	3477	16.9	0.48	2.97	0.09	18.50	0.53	2675	76.93
18037	4.94	0.07	1.41	0.33	6.64	1.54	31.10	1.49	30.11	18037	3597	39.2	1.09	5.13	0.14	17.54	0.49	2444	67.94
18040	4.79	0.03	0.73	0.24	5.11	1.78	37.06	2.14	44.64	18040	3657	14.6	0.40	10.83	0.30	21.17	0.58	3024	82.70
18043	4.81	0.02	0.44	0.29	5.96	1.69	35.15	2.03	42.27	18043	3597	17.4	0.48	10.75	0.30	26.36	0.73	2914	81.02
18045	5.01	0.03	0.56	0.24	4.75	1.83	36.57	2.01	40.20	18045	3777	17.8	0.47	5.57	0.15	21.31	0.56	2907	76.98
18048	5.08	0.08	1.65	0.28	5.50	1.92	37.67	2.10	41.24	18048	3777	67.3	1.78	10.00	0.26	31.49	0.83	3012	79.76
18050	5.13	0.07	1.36	0.27	5.31	1.50	29.27	2.44	47.52	18050	3777	21.0	0.56	3.25	0.09	22.77	0.60	3039	80.45

Al	Al	Al	Ratios	Al	Ratios	Al	Ratios	Al	Ratios
Sample ID	%								
18001	8.9	0.1	1.3	0.2	2.8	1.6	18.2	6.5	73.0
18004	8.9	0.1	1.7	0.2	2.8	1.8	20.8	6.0	67.7
18006	8.8	0.1	0.9	0.2	2.8	1.8	20.4	6.0	68.3
18009	8.9	0.1	1.5	0.4	4.0	2.0	22.2	5.7	64.0
18011	8.7	0.1	1.4	0.5	5.8	2.2	24.8	5.2	60.0
18015	8.9	0.1	1.4	0.9	10.6	2.5	28.7	4.6	52.1
18016	8.6	0.1	1.6	1.1	12.6	2.3	26.7	4.4	50.5
18017	8.0	0.1	1.8	1.0	12.7	1.9	23.4	4.4	54.8
18018	7.7	0.1	1.7	0.7	9.7	1.5	19.6	4.5	58.6
18020	7.7	0.1	1.7	0.7	9.1	1.4	18.1	4.0	52.2
18021	7.4	0.2	2.6	0.7	9.0	1.5	20.2	4.7	63.3
18023	7.3	0.1	0.9	0.8	10.5	1.5	20.1	3.8	52.6
18024	7.2	0.2	2.1	0.7	9.4	1.6	22.4	4.0	56.1
18025	7.4	0.1	1.8	0.6	8.5	1.7	23.2	3.9	52.5
18026	7.5	0.1	1.5	0.4	5.4	1.7	23.3	3.8	51.2
18027	8.3	0.1	1.3	0.6	7.0	1.8	21.3	6.4	77.1
18029	8.2	0.1	0.8	0.1	1.8	1.2	14.3	5.6	69.0
18031	8.4	0.1	0.7	0.1	1.2	1.2	14.3	5.9	70.0

18033	8.1	0.1	1.4	0.1	1.4	1.3	15.8	5.9	72.3
18037	8.1	0.2	2.1	0.2	1.9	1.3	15.6	4.6	56.4
18040	8.2	0.1	1.5	0.1	1.3	1.4	17.6	5.7	69.9
18043	7.8	0.1	0.9	0.1	1.6	1.4	17.7	5.5	70.3
18045	7.9	0.1	1.1	0.1	0.9	1.4	18.1	5.5	69.2
18048	7.7	0.2	2.9	0.1	1.2	1.5	19.3	5.4	70.3
18050	7.6	0.1	1.1	0.1	1.1	1.1	13.8	5.7	74.9

[1] The concentrations is relative to the bulk sample weight; [2] The ratios is relative to the bulk sample concentrations

Supplementary Table S3. Concentrations of Ti, Fe, Mn, and Mo in two sequential extraction phases (phosphate phase and Fe-Mn (hydro) oxide phase), along with $\delta^{98}Mo$ values in the Fe-Mn (hydro)oxide phase for the GC112 and XT19 cores.

	**Phosphate phase**										**Fe and Mn (hydro) oxide phase**											
	**Ti**	**Al**	**Mn**	**Fe**	**Mo**	**Ti**	**Al**	**Mn**	**Fe**	**Mo**	**δ^{98}Mo**	**2SE**	**Ti**	**Al**	**Mn**	**Fe**	**Mo**	**Ti**	**Al**	**Mn**	**Fe**	**Mo**
	μg/g[1]					%[2]					‰		μg/g					%				
CC112 core																						
18004	4.5	287	307	285	6.4	0.07	0.32	3.91	0.48	26.5	-0.70	0.039	77.5	17094	7417	33697	13.9	1.3	19.3	94.5	56.7	57.9
18006	4.9	254	334	279	9.3	0.09	0.29	3.51	0.43	27.1	-0.86	0.029	64.3	16489	8932	32825	18.6	1.1	18.7	93.9	51.1	54.3
18017	8.6	216	251	326	10.7	0.18	0.27	2.26	0.62	18.2	-0.49	0.034	54.3	19105	9646	20951	33.9	1.2	24.0	86.8	40.1	57.7
18018	24.3	376	262	2467	21.4	0.57	0.49	2.14	5.01	29.6	-0.52	0.038	45.0	14807	9241	15295	35.3	1.1	19.2	75.5	31.1	48.8
18020	12.0	177	227	371	10.1	0.32	0.23	1.76	0.81	13.2	-0.46	0.036	37.4	12514	8971	13528	35.9	1.0	16.3	69.5	29.7	47.2
18024	21.3	275	227	1768	14.6	0.59	0.38	1.88	4.01	21.3	-0.38	0.031	52.9	14886	9680	14647	40.2	1.5	20.7	80.0	33.2	58.7
18025	12.4	198	194	391	8.4	0.33	0.27	1.42	0.81	11.2	-0.38	0.034	56.7	15869	11751	17004	45.8	1.5	21.6	85.8	35.2	60.7
18026	9.2	186	153	349	6.2	0.28	0.25	0.92	0.75	6.81	-0.26	0.043	36.8	13588	15568	16825	66.9	1.1	18.1	93.7	36.2	73.3
18027	7.6	221	139	312	6.8	0.22	0.27	0.71	0.67	5.92	-0.28	0.029	20.0	11238	16238	15214	70.9	0.6	13.6	82.3	32.7	61.5
18048	8.1	157	126	319	4.7	0.24	0.23	0.49	0.67	3.71	0.04	0.050	11.5	7117	12746	11263	41.3	0.3	9.2	58.1	22.1	44.2
18050	5.3	130	118	234	3.8	0.21	0.20	0.58	0.63	5.00	0.02	0.030	13.8	7397	13873	12539	44.2	0.4	9.7	63.8	24.4	49.7
XT19 core																						
XT19-8	15	970	761	1590	1.17	0.28	0.94	10.2	2.29	5.49	-0.47	0.07	1280	34602	6324	54192	18.0	24.9	33.6	84.6	77.9	84.6
XT19-36	17	830	602	1689	1.45	0.34	0.77	7.90	2.30	5.06	-0.23	0.05	1360	35118	6709	55211	25.5	27.2	32.5	88.0	75.2	88.8
XT19-57	11	713	663	1183	1.12	0.21	0.71	7.42	1.67	2.99	-0.10	0.06	1314	34857	7708	57223	31.9	25.6	34.5	86.3	80.6	85.5
XT19-78	8	675	578	1263	1.19	0.17	0.66	7.64	1.78	3.42	0.10	0.04	1165	33803	7141	57044	30.8	23.6	33.0	94.3	80.4	88.3

XT19-113	9	677	681	1147	1.09	0.19	0.68	8.40	1.69	3.14	0.08	0.04	1153	32028	7223	54310	30.8	23.7	32.3	89.2	80.0	88.8
XT19-163	12	676	792	1311	1.14	0.26	0.69	8.74	1.88	2.89	-0.03	0.05	1332	33541	8287	56992	36.2	27.9	34.0	91.4	81.9	91.4
XT19-176	12	1532	737	1191	0.96	0.25	1.56	8.34	1.71	2.82	-0.06	0.07	1349	34555	8615	58720	31.6	28.6	35.2	97.5	84.1	92.4
XT19-183	16	658	726	1266	0.98	0.35	0.66	8.41	1.80	2.89	0.16	0.04	1342	35075	8080	57380	29.9	28.9	35.4	93.5	81.5	88.4
XT19-218	10	716	636	1287	1.33	0.21	0.73	8.06	1.82	3.87	-0.06	0.05	1447	38611	7044	58415	30.0	29.5	39.5	89.2	82.4	87.3

[1] The concentrations is relative to the bulk sample weight; [2] The ratios is relative to the bulk sample concentrations.

Supplementary Table S4. Fluxes and isotopic compositions of dissolved Mo into and out of the oceans based on the updated oxic sediment sink

		Flux ($\times 10^8$ mol yr^{-1})	Flux range ($\times 10^8$ mol yr^{-1})	Fraction	δ^{98}Mo (‰)	δ^{98}Mo (‰) range	$F_{in} * \delta_{in}$	References
**Inputs**	River	3.1		0.92	0.80		0.74	24, 25
	Low T Hydrothermal	0.26		0.08	0.80		0.06	26, 27
	Total	*3.36*		*1.00*			*0.80*	
		Flux ($\times 10^8$ mol yr^{-1})	Flux range ($\times 10^8$ mol yr^{-1})	Fraction	δ^{98}Mo (‰)		$F_{out} * \delta_{out}$	References
**Outputs**	**Oxic**	**1.52**	**0.99-1.65**	**0.45**	**−0.09**	**−1.0–−0.09**	**-0.04**	9, 28, 29, 30; this study
	Anoxic margin	1.45	0.8-1.65	0.43	1.61	−0.09–1.8	0.70	19, 29, 30
	Euxinic	0.28	0.20-0.50	0.09	1.90	1.8–2.3	0.16	19, 29, 30
	High T hydrothermal	0.1	0.04-0.17	0.03	-0.5		-0.01	19, 29, 30
	Total	*3.36*		*1.00*			*0.80*	

Supplementary References

1. Murray RW, Leinen M. Scavenged excess aluminum and its relationship to bulk titanium in biogenic sediment from the central equatorial Pacific Ocean. *Geochim Cosmochim Ac* **60**, 3869-3878 (1996).

2. Wei G, Liu Y, Li X, Chen M, Wei W. High-resolution elemental records from the South China Sea and their paleoproductivity implications, *Paleoceanography* **18,** (2003).

3. Wei G, Liu Y, Li X-h, Shao L, Fang D. Major and trace element variations of the sediments at ODP Site 1144, South China Sea, during the last 230 ka and their paleoclimate implications. *Palaeogeography, Palaeoclimatology, Palaeoecology* **212**, 331-342 (2004).

4. Rudnick, R.L. and Gao, S. (2014) Composition of the Continental Crust. In: Holland, H.D. and Turekian, K.K., Eds., Treatise on Geochemistry, Elsevier, Oxford, 1-51. https://doi.org/10.1016/B978-0-08-095975-7.00301-6.

5. Greber ND, Puchtel IS, Nägler TF, Mezger K. Komatiites constrain molybdenum isotope composition of the Earth's mantle. *Earth Planet Sc Lett* **421**, 129-138 (2015).

6. Liang YH, et al. Molybdenum isotope fractionation in the mantle. Geochim Cosmochim Ac **199**, 91-111 (2017).

7. Voegelin AR, Pettke T, Greber ND, von Niederhäusern B, Nägler TF. Magma differentiation fractionates Mo isotope ratios: Evidence from the Kos Plateau Tuff (Aegean Arc). *Lithos* **190**, 440-448 (2014).

8. Yang J, Barling J, Siebert C, Fietzke J, Stephens E, Halliday AN. The molybdenum isotopic compositions of I-, S-and A-type granitic suites. *Geochim Cosmochim Ac* **205**, 168-186 (2017).

9. Bertine KK, Turekian KK. Molybdenum in Marine Deposits. *Geochim Cosmochim Ac* **37**, 1415-1434 (1973).

10. Bi DJ, et al. Geochemical and mineralogical characteristics of deep-sea sediments from the western North Pacific Ocean: Constraints on the enrichment processes of rare earth elements. *Ore Geol Rev* **138**, 104318 (2021).

11. Brucker RLP, McManus J, Severmann S, Berelson WM. Molybdenum behavior during early diagenesis: Insights from Mo isotopes. *Geochemistry Geophysics Geosystems* **10**, Q06010 (2009).

12. Chen S, et al. Extremely light molybdenum isotope signature of sediments in the Mariana Trench. *Chem Geol* **605**, 120959 (2022).

13. Tanaka E, Mimura K, Nakamura K, Ohta J, Yasukawa K, Kato Y. Rare-Earth Elements in Deep-Sea Sediments in the South Pacific Gyre: Source Materials and Resource Potentials. *Geochemistry Geophysics Geosystems* **24**, e2022GC010681 (2023).

14. Tanaka E, et al. Chemostratigraphy of deep-sea sediments in the western North Pacific Ocean: Implications for genesis of mud highly enriched in rare-earth elements and yttrium. *Ore Geol Rev* **119**, 103392 (2020).

15. Deng K, Yang S, Guo Y. A global temperature control of silicate weathering intensity. *Nat Commun* **13**, 1781 (2022).

16. Guo L, et al. Acceleration of phosphorus weathering under warm climates. 10, eadm7773 (2024).

17. Chen X, et al. Rise to modern levels of ocean oxygenation coincided with the Cambrian radiation of animals. *Nat Commun* **6**, 7142 (2015).

18. Wei G-Y, et al. Global marine redox evolution from the late Neoproterozoic to the early Paleozoic constrained by the integration of Mo and U isotope records. *Earth-Sci Rev* **214**, 103506 (2021).

19. Kendall B, Dahl TW, Anbar AD. The Stable Isotope Geochemistry of Molybdenum. *Non-Traditional Stable Isotopes* **82**, 683-732 (2017).

20. Wang Z, et al. “Revised Oceanic Mo Isotope Budget from Deep-Sea Pelagic Sediments.” Mendeley

Data, V5 (2025). https://data.mendeley.com/datasets/fy4nppvx6v/5.

21. Schlitzer, R., eGEOTRACES - Electronic Atlas of GEOTRACES Sections and Animated 3D Scenes. (2021), http://www.egeotraces.org. 2021.

22. Bai J, et al. Stable Nd isotopic fractionation in REY-rich deep-sea sediments. *Earth Planet Sc Lett* **652**, 119197 (2025).

23. Zhang GL, et al. Balancing the oceanic Zn isotope budget: The key role of deep-sea pelagic sediments. *Geology* **52**, 789-793 (2024).

24. Miller CA, Peucker-Ehrenbrink B, Walker BD, Marcantonio F. Re-assessing the surface cycling of molybdenum and rhenium. *Geochim Cosmochim Ac* **75**, 7146-7179 (2011).

25. Revels BN, Rickli J, Moura CAV, Vance D. The riverine flux of molybdenum and its isotopes to the ocean: Weathering processes and dissolved-particulate partitioning in the Amazon basin. *Earth Planet Sc Lett* **559**, 116773 (2021).

26. McManus J, Nägler TF, Siebert C, Wheat CG, Hammond DE. Oceanic molybdenum isotope fractionation: Diagenesis and hydrothermal ridge-flank alteration -: art. no. 1078. *Geochemistry Geophysics Geosystems* **3**, 1078 (2002).

27. Wheat CG, Mottl MJ, Rudnicki M. Trace element and REE composition of a low-temperature ridge flank hydrothermal spring. *Geochim Cosmochim Ac* **66**, 3693-3705 (2002).

28. Morford JL, Emerson S. The geochemistry of redox sensitive trace metals in sediments. *Geochim Cosmochim Ac* **63**, 1735-1750 (1999).

29. Poulson RL, Siebert C, McManus J, Berelson WM. Authigenic molybdenum isotope signatures in marine sediments. *Geology* **34**, 617-620 (2006).

30. Siebert C, Nägler TF, von Blanckenburg F, Kramers JD. Molybdenum isotope records as a potential

new proxy for paleoceanography. *Earth Planet Sc Lett* **211**, 159-171 (2003).
