# Supplementary material for: Revised oceanic molybdenum isotope budget from deep-sea pelagic sediments
Source: Nat Commun. 2025 Nov 18;16:10086. doi: 10.1038/s41467-025-65006-5 (PMC12627635; doi:10.1038/s41467-025-65006-5)
Supplement: Supplementary file 2 — Description of Additional Supplementary Files [file 41467_2025_65006_MOESM2_ESM.pdf]

Description of Additional Supplementary Files:

Supplementary Datasets 1: Published data on Mo content and associated geochemical parameters in global pelagic sediments.

Supplementary Datasets 2: Published data on Mo isotopes and associated geochemical parameters in global pelagic sediments.
